# Soybean (*Glycine max* L. Merr.) Sprouts Germinated under Red Light Irradiation Induce Disease Resistance against Bacterial Rotting Disease

**DOI:** 10.1371/journal.pone.0117712

**Published:** 2015-02-13

**Authors:** Radhika Dhakal, Euiho Park, Se-Weon Lee, Kwang-Hyun Baek

**Affiliations:** 1 School of Biotechnology, Yeungnam University, Gyeongsan, Gyeongbuk, Republic of Korea; 2 International Technology Cooperation Center, Rural Development Administration, Jeonju, Republic of Korea; Soonchunhyang University, KOREA, REPUBLIC OF

## Abstract

Specific wavelengths of light can exert various physiological changes in plants, including effects on responses to disease incidence. To determine whether specific light wavelength had effects on rotting disease caused by *Pseudomonas putida* 229, soybean sprouts were germinated under a narrow range of wavelengths from light emitting diodes (LEDs), including red (650–660), far red (720–730) and blue (440–450 nm) or broad range of wavelength from daylight fluorescence bulbs. The controls were composed of soybean sprouts germinated in darkness. After germination under different conditions for 5 days, the soybean sprouts were inoculated with *P. putida* 229 and the disease incidence was observed for 5 days. The sprouts exposed to red light showed increased resistance against *P. putida* 229 relative to those grown under other conditions. Soybean sprouts germinated under red light accumulated high levels of salicylic acid (SA) accompanied with up-regulation of the biosynthetic gene *ICS* and the pathogenesis- related (PR) gene *PR-1*, indicating that the resistance was induced by the action of SA via *de novo* synthesis of SA in the soybean sprouts by red light irradiation. Taken together, these data suggest that only the narrow range of red light can induce disease resistance in soybean sprouts, regulated by the SA-dependent pathway via the *de novo* synthesis of SA and up-regulation of PR genes.

## Introduction

Soybean (*Glycine max* L. Merr.) is one of the most important crops in terms of providing oil and protein [1]; accordingly, soybean has been used as a model system for the seed developmental process [2]. The consumption of soy food exerts high benefits on human health including reduced incidence of coronary heart disease, reduced risk of breast and prostate cancers, improved bone health and relief of menopausal symptoms [3]. Soybeans are utilized in a variety of foods including soymilk, soy yoghurt, tofu, miso, soy sauce, soy flour, soy cheese, green and dried soybeans, soybean sprouts, and other fermented food products [4]. Additionally, soybean sprouts are commonly consumed in Northeast Asian countries including Korea, China and Japan as vegetables in soups, salads and side dishes [5].

Any type of reduction in soybean yield mainly occurs during the sprout stage of development [6]. Different diseases cause loss of soybean production, and causal organisms responsible for such losses include fungi and bacteria such as *Rhizoctonia* sp., *Pseudomonas* sp., *Phytopthora* sp. and *Bradyrhizobium japonicum* [1,7–9]. Furthermore, sprouts have been shown to be the means of transmission of a number of food borne outbreaks of infection [6,10,11]. These occurrences of infection integrated salmonella poisoning and *Escherichia coli* 0157 infection, and implicated all kinds of seed sprouts, including alfalfa, clover, cress, mung bean, radish and soybean [6,11]. Thus, microbial infection of soybean sprouts has large negative impacts on soybean production and the soybean sprout industry.

Light plays an essential role in plant growth and development together with host defensive mechanisms. Subjecting plants to specific wavelengths of light such as ultraviolet (UV) and red light can induce plants to develop higher levels of disease resistance against pathogens. UV-C irradiation leads to accumulation of phytoalexin hydroxyphaseollin, which helps soybean plants develop resistance against *P*. *megasperma* var. s*ojae* [12]. UV irradiation also facilitates accumulation of the phytoalexins sakuranetin and oryzalexin F in rice leaves, which might help increase resistance against microbial pathogens [13,14]. Red light treatment of pepper, pumpkin, and tomato seedlings led to development of resistance against *P*. *capsici* [15]. Furthermore, systemic disease resistance against root-knot nematode *Meloidogyne javanica* and a bacterial disease, *P*. *syringae* pv. Tomato DC 3000, was induced by pretreatment of *Arabidopsis* with red light [16].

Salicylic acid (SA) is a small phenolic compound produced by both prokaryotes and eukaryotes [17]. Biotic and abiotic stimuli increase the endogenous levels of SA in plants, which helps induce the defense mechanism [17]. SA-mediates plant immune response to systemic acquired resistance (SAR), which limits the growth of biotrophic and necrotrophic virulent pathogens and favors long-term protection against a broad spectrum of microorganisms [18–21]. *P*. *fluorescens* WCS417r triggered SAR by accumulating endogenous SA in radish but not in *Arabidopsis* which induced systemic resistance through SA-independent signaling pathway [22,23]. The endogenously increased SA describes the state of SAR by inducing the expression levels of pathogenesis-related (PR) genes, such as *PR*-*1*, *PR*-*2* and *PR*-*5*, which are considered to be the effector genes for SAR [24,25]. SA does not always induce resistance to pathogens, shown in the suppressed resistance by SA addition in the induced resistance in the broad beans by red light against *Botrytis cinerea* [26]. The SA-dependent defense system is working specifically based on the relation between a plant host species and a pathogen species. In tomato plants, SA-dependent defense pathway induced resistances to *Botrytis cinerea* but not to *Oidium neolycopersici*, however, in the tobacco it induced resistance to *O*. *neolycopersici* but not to *B*.*cinerea* [27]. Therefore, plant defense responses activated by SA-dependent pathway depend on a specific host-pathogen system, not on a commonly shared system even differed in a closely related host-pathogen relation.

Jasmonic acid (JA) and SA are essential plant hormones in plant-pathogen defense signaling pathway regulating induction of *PR* genes [28]. These two plant hormones signaling pathways are mutually antagonistic or show no modulation in one another induction [29,30]. Silver leaf whitefly infested *Arabidopsis* leaves induced the SA-regulated *PR-1* gene transcripts, but no or very low expression of *PDF1*.*2*, a JA-regulated gene marker [30]. SA treatment blocked the biosynthesis of JA in tomato leaves and production of JA was inhibited by SA application in wounded tobacco plants [31,32]. Exogenous application of SA induced the expression of acidic *PR* genes, but the expression of the genes was inhibited by JA application. In contrast, JA application induced the expression of basic *PR* genes, which expression was hampered by SA treatment [28]. All evidence indicates that SA and JA exert antagonistic effects on plant-pathogen defense signaling.

Recently, the availability of light emitting diodes (LEDs) has made it possible to study the effects of narrow ranges of light wavelength on plant growth and development. Previously, the roles of a certain wavelength on the physiology of plants were studied by employing light filters. However, LED technology has enabled replacement of filters with LEDs that emit single wavelengths of light. Use of LEDs as light sources can easily make the emitting wavelength and light intensity selectable; therefore, LED application to plant experiments has resulted in a great increase in accumulation of knowledge regarding the effects of individual light wavelengths on plant growth and development [33].

In this study, we irradiated soybean sprouts with a narrow range of light wavelength to determine (i) whether soybean sprouts germinated under continuous irradiation with red light gained resistance against *Pseudomonas putida* 229, a rotting bacterium of soybean sprouts and (ii) the underlying mechanism for the induced resistance, especially for SA-mediated resistance.

## Materials and Methods

### Germination of soybean sprouts under irradiation with different wavelengths of light

Seeds of soybean [*Glycine max* (L.) Merr. cv. Pungsan] were provided by Dr. Euiho Park, who had preserved soybean stocks in a seed storage room maintained with low moisture and low temperature. The soybeans used in this experiment were harvested from the field of Yeungnam University in the Fall of 2012. Seeds were washed in double distilled water (ddH_2_O), sterilized by dipping and shaking in 70% ethanol for 30 s, and then rinsed with ddH_2_O at least five times. Next, the sterilized seeds were soaked in ddH_2_O for 4 h at 25°C to initiate germination, after which the surface water was removed gently by hand shaking and the seeds were placed on wet tissue papers of uniform thickness in a plastic wicker tray. The trays with soybean seeds were placed in separate sectors of a chamber at 25°C and 50% humidity. Each sector was then irradiated with different light sources, including LEDs emitting light at 440, 660, or 730 nm (blue, red, and far-red, respectively) and daylight fluorescence (DLF) bulbs (three band lamp, 11W, Alim Industry, Republic of Korea). The red, far-red, blue and fluorescent light sources had light intensities of 46.10, 2.19, 35.01 and 10.85 μM photons /m^2^s, respectively. The control was composed of soybean seeds germinated under the same conditions without any light treatment. The soybean seeds were grown for 5 days while providing the proper amount of ddH_2_O daily to prevent the seeds from drying out.

### Bacterial inoculation

A single colony of *P*. *putida* 229, a pathogenic bacteria that causes soybean sprout rotting, was grown in nutrient broth (NB) media (Difco Nutrient Broth, Becton, Dickinson and Company, Sparks, USA) at 25°C for 2 days with shaking at 100 rpm. After removing the root tips (3cm from the end) from soybean sprouts grown for 5 days with a sterile scalpel, the remaining parts were inoculated by immersion in *P*. *putida* 229 culture adjusted to an OD_600_ of 1.0 for 8 h. The soybean sprouts were recovered from the culture solution, after which the culture drops on the surface were removed by gentle hand shaking, and samples were incubated on wet-tissue paper at 25°C in darkness for 5 days, during which time they were observed for disease incidence. The disease incidence was calculated by dividing the number of the infected soybean hypocotyls by the total number of hypocotyls inoculated in each treatment. Fifteen hypocotyls were inoculated with *P*. *putida* 229 in each treatment, and those experiments were repeated four times.

To prepare the samples for SA and JA analysis and gene expression, 5 day old soybean sprouts inoculated with *P*. *putida* 229 were subjected to the following treatments: control (0 h inoculation + 0 h incubation), 6 h treatment (3 h inoculation + 3 h incubation), 16 h treatment (8 h inoculation + 8 h incubation) and 32 h treatment (8 h inoculation + 24 h incubation). Only hypocotyls were collected and used for the extraction of SA, JA and total RNA.

### Analysis of the contents of SA and JA

The extraction of SA from the soybean hypocotyls was conducted according to the method described by Marek et al. [34], with slight modification. Briefly, 0.5 g of hypocotyls were ground with a mortar and pestle, mixed with 3 mL of 90% methanol, and then centrifuged at 14,000g at 4°C for 10 min, after which the supernatant was collected. The pellets were re-extracted with 1.5 mL of 100% methanol, after which the supernatant was combined with the previously collected supernatant. The combined supernatant samples were then concentrated to a final volume of around 250 μL using a speed vacuum (miVac DUO concentrator, New York, USA), then resuspended in 1 mL of hydrolysis buffer (0.1 M sodium acetate buffer, pH 5.5).

Next, the mixture was split into two equal volumes and analyzed for free SA and glucose-conjugated SA (salicylic acid 2-*O*-β-D-glucoside, SAG) in two separate tubes. To determine the amount of SAG, 10 units of β- glucosidase (Sigma-Aldrich, St. Louis, MO USA) were added to the tube. Following incubation at 37°C for 1.5 h, 625 μL of 10% TCA was added to both tubes, after which the samples were centrifuged at 14,000 g and 4°C for 10 min. The supernatant was then transferred to a fresh tube and mixed with 1 mL extraction solvent composed of ethylacetate: cyclohexane (1:1 ratio). Only the top organic phase was transferred into a fresh tube, after which it was concentrated to a final volume of ∼125 μL using a speed vacuum. Next, the residue was resuspended in 0.5 mL of 0.2 M sodium acetate buffer (pH 5.5), after which it was centrifuged at 14,000 g at 4°C for 10 min. The supernatant was subsequently removed and filtered using a 0.45 μm nylon filter (Chemco Scientific, Japan).

Extraction of JA was performed according to the method described by Muller and Bosch [35] with minor modifications. In brief, 100 mg of frozen soybean hypocotyls were ground with a motor and pestle using liquid nitrogen, and mixed with 2 mL of extraction solvents composed of methanol: isopropanol (20:80 ratio) with 1% glacial acetic acid. The solvent-mixed samples were then sonicated at 4–10°C for 30 min and centrifuged at 10,000 g at 4°C for 10 min. The supernatant was collected in a glass vial, and the residue was re-extracted with 1 mL of extraction solvent by following the same procedure. The newly collected supernatant was added into the original supernatant in the same glass vial, and dried under nitrogen gas stream. The dried samples were mixed with 500 μL of methanol and filtered using the 0.45 μm nylon filter (Chemco Scientific, Japan).

After the extraction of SA and JA, both the compound amount were determined using an HPLC with a Denali C18 120A 5μ, 150mm × 4.6 mm column (Grace Davison Discovery Sciences, Illinois, USA) and an UV detector (YL9100, Young-Lin, Republic of Korea). The mobile phase used for SA and JA analysis was 0.2 M sodium acetate buffer (pH 5.5) in 10% methanol at a flow rate of 0.80 mL mˉ^1^ and acetonitrile: water (25:75) with 0.1% trifluoroacetic acid (TFA) at a flow rate of 1 mL mˉ^1^, respectively. The amount of SA and JA were determined by comparing the area for the corresponding peaks with the standard curve drawn using different concentrations of free SA (Duchefa Biochemie, Netherlands) and JA (Sigma-Aldrich, St. Louis, USA).

### RNA extraction and semi-quantitative RT-PCR

Total RNA was isolated from the hypocotyls under the same conditions as the SA and JA extraction using the Tri-Reagent solution (Molecular Research Center, Inc., Ohio, USA) according to the manufacturer’s protocol. Next, 50 ng of total RNA were used for semi-quantitative RT-PCR (TITANIUM one step RT-PCR kit, Takara Bio Inc., Japan) on a PCR machine (XP Thermal Cycler, BIOER, Japan). The primers for the candidate genes were designed using the Primer3 (v.0.4.0) program (http://bioinfo.ut.ee/primer3-0.4.0/) [36,37]. The primers for the genes in the biosynthesis of SA and JA and for the PR genes are summarized in Table 1. The genes selected for the SA synthesis were *phenylalanine ammonium lyase* (*PAL*) and *isochorismate synthase* (*ICS*). The genes for the JA synthesis included *acyl-CoA oxidase* (*ACX*) and *3-ketoacyl-CoA thiolase* (*KAT*). The *PR-1* and *PR-4* genes were selected for the SA and JA- dependent PR genes, respectively. *Actin* gene was used as a reference to verify equal amounts of total RNA. Semi-quantitative RT-PCR was conducted under the following reaction conditions: initial denaturation at 95°C for 5 min, followed by 30 cycles of 95°C for 20 s, 50–54°C for 30 s and 72°C for 40 s, and then final extension at 72°C for 5 min. The PCR products were visualized by electrophoresis on 1.2% agarose gel.

**Table 1:** Primers used for PCR and their amplicon sizes.

Genes (NCBI accession no.)	Primer pairs	Amplicon size (bp)
*Isochorismate synthase* (*ICS*) (AW596452.1)	Forward; 5’–CAACAGAAGAGGCACAACTT– 3’	215
	Reverse; 5’–GAGTTCTAGCTCATCCCACTC– 3’	
*Phenylalanine ammonium lyase* (*PAL*) (X52953.1)	Forward; 5’ –GGAGTCTCTATGGACAACACAC –3’	194
	Reverse; 5’ –TGGAGTTCAGAGCAGTAAGAAG– 3’	
*Acyl-CoA Oxidase* (*ACX*) (NM_001250062.1)	Forward; 5’ –CTGGTCTTTCTATCACTGGAAG– 3’	216
	Reverse; 5’–CATCACTTCCATAGTCAGGTTC– 3’	
3-*ketoacyl-CoA thiolase* (*KAT*) (AY383736.1)	Forward; 5’– CAGATAGAGGAATTGAGTGCAG –3’	198
	Reverse; 5’– GCCTATTCACACGATCTACTGT –3’	
*Pathogenesis- related*1 (*PR*-1) (AF136636.1)	Forward; 5’ –TGATGTTGCCTACGCTCAAG –3’	137
	Reverse; 5’ –AAGCAGCAACCGTATCATCC– 3’	
*Pathogenesis- related*4 (*PR*-4) (Z11977.1)	Forward; 5’ –GCTTGCGGGTGACAAATAC– 3’	96
	Reverse; 5’ –ACACTCCCACGTCCAAATC– 3’	
Actin (U60500.1)	Forward; 5’ –GAGAGAGGATACTCCTTCAGC –3’	204
	Reverse; 5’ –GAACAGTACTTCTGGGCAAC –3’	

### Statistical analysis

All numeric data represent the means of three samples ± the standard deviation (SD). The variance of the sample data was identified by Duncan’s test using the Statistical analysis software (SAS) version 9.1 (SAS Inc., Cary, NC, USA).

## Results

### Growth of soybean sprouts under different germinating conditions

**Fig 1:**
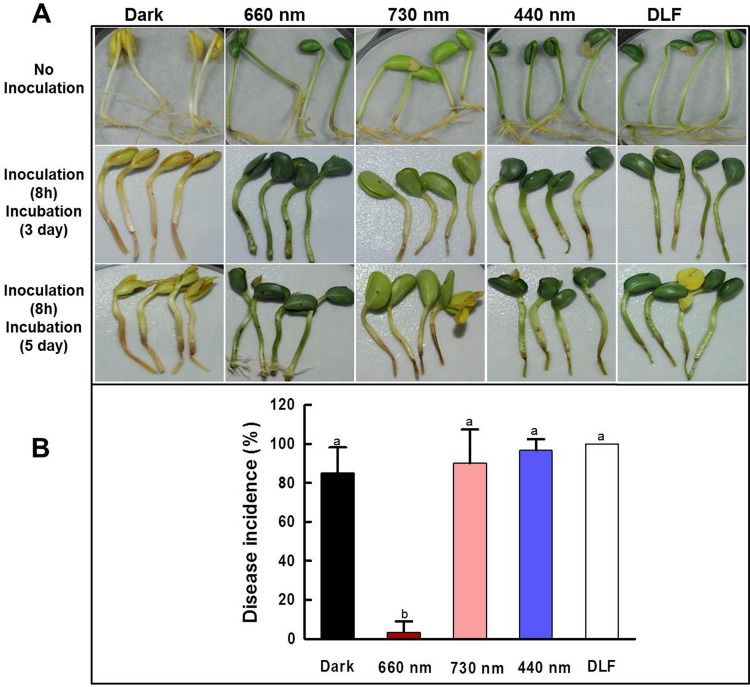
Effects of different wavelengths on the growth of soybean sprouts and disease susceptibility to *Pseudomonas putida* 229. Soybean sprouts were germinated for 5 days under different conditions. (A) The soybean sprouts germinated in darkness, red light, far-red light, blue light and fluorescence daylight. The sprouts tips were cut, inoculated in bacterial culture for 8 h, and then incubated for 5 days in darkness. The disease symptoms were observed after 3 and 5 days of incubation in darkness. (B) Comparison of disease incidence in soybean sprouts germinated in darkness and under different light irradiation for 5 days. The experiments were repeated four times. The different letter indicates a significant difference (*P*<0.01).

All soybean seeds germinated under the aforementioned conditions; however, they developed different colors and hypocotyl growth (Fig. 1A). The sprouts germinated under red, blue and white light were dark green, while those germinated under far-red light were light green (Fig. 1A). The sprouts germinated in darkness had yellow seeds and white hypocotyls due to the lack of chlorophylls in the absence of photosynthesis. Additionally, the sprouts germinated under far-red light had shorter hypocotyls than those grown under other conditions (Fig. 1A).

### Red light-induced resistance of soybean sprouts to *P*. *putida* 229

Soybean sprouts germinated under different conditions were incubated in darkness for a total of 5 days after inoculation (DAI) of *P*. *putida* 229 to measure the disease incidence. Except for sprouts germinated under red light, other sprouts germinated in darkness and under other wavelengths of light rotted at 5 DAI with *P*. *putida* 229 (Fig. 1A). The soybean sprouts germinated under red light had not developed any symptoms of disease at 3 DAI (Fig. 1A), and had fully recovered from the disease and grew adventitious root at 5 DAI (Fig. 1A). The soybean sprouts germinated under red light had only 3.3% disease incidence, which was significantly very lower than those germinated in darkness or under other wavelengths (Fig. 1B). The soybean sprouts germinated in darkness and under blue light, far red light and daylight fluorescence had 85%, 96.7%, 90% and 100% disease incidence, respectively. (Fig. 1B).

### Expression analysis of genes in the biosynthetic pathway of SA and JA

After observation of disease resistance in soybean sprouts, we examined the expression levels of genes in the pathway of SA and JA biosynthesis to determine which plant hormone was responsible for the increased resistance in soybean sprouts. Furthermore, the expression levels of the *PR-1* and *PR-4* genes induced by SA and JA, respectively, were analyzed to confirm that the defense molecule was induced by red light irradiation.

**Fig 2:**
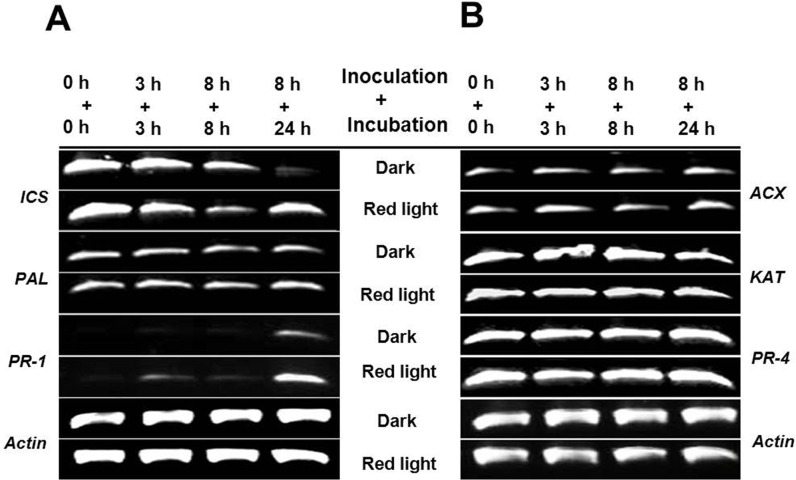
Expression profile of genes in the biosynthetic pathways of (A) Salicylic acid and (B) jasmonic acid, and their pathogenesis-related (PR) genes. Abbreviation: *ICS*, *isochorismate synthase*; *PAL*, *phenylalanine ammonium lyase; ACX*, *acyl-CoA oxidase*; *KAT*, *3-ketoacyl-CoA thiolase*. The actin gene was used as the reference gene for equal usage of total RNA.

The biosynthesis of SA takes place from chorismic acid via the enzymatic reaction of ICS or PAL Wildermuth et al. [38]. From chorismic acid, the ICS or PAL is the committed step for further SA biosynthesis [30], therefore, the genes of *ICS* and *PAL* were selected to determine the expression levels for the *de novo* synthesis of SA by the infection of *P*. *putida* 229. When the expression levels of the *ICS* gene were measured, the *ICS* gene appeared to be highly expressed in soybean sprouts grown under red light (0 h + 0h) (Fig. 2A); therefore, the gene was responsible for the higher levels of SA in sprouts grown under red light. The expression levels in the soybean sprouts grown in darkness or under red light decreased after being incubated with *P*. *putida* 229 (3 h + 3 h and 8 h + 8 h), but returned to the original expression levels in soybean sprouts grown under red light after 24 h (Fig. 2A). Expression of the *PAL* gene did not differ significantly among soybean sprouts grown in darkness or under red light, or in response to infection with *P*. *putida* 229 (Fig. 2A). To confirm that the increased levels of SA affected expression of the *PR* gene, we measured the expression level of the *PR-1* gene, which is a downstream marker of SA accumulation. After incubation for 24 h (8 h + 24 h), the gene was up-regulated to a greater degree in soybean sprouts grown under red light than in those grown in darkness, but gene expression was not detected in un-infected sprouts (0 h + 0 h) (Fig. 2A).

In the JA biosynthesis, 3-oxo-2-[2’(*Z*)-pentenyl]-cyclopentane-1-octanoic acid (OPC8:0) is converted to 2-trans-enoyl-CoA by acyl-CoA oxidase (ACX), then synthesized to JA by 3-ketoacyl-CoA thiolase (KAT) [39], therefore, the two enzymes were selected to determine the gene expression. Both *ACX* and *KAT* genes were expressed in soybeans grown in darkness and under red light, but their expression levels did not change in response to *P*. *putida* 229 infection (Fig. 2B). Concordant with the data describing genes involved in the biosynthesis of JA, the expression level of the *PR-4* gene induced by high JA content did not change, but was expressed at high levels with or without *P*. *putida* 229 infections (Fig. 2B).

### Free- and conjugated- SA accumulation in soybean sprouts irradiated with red light

**Fig 3:**
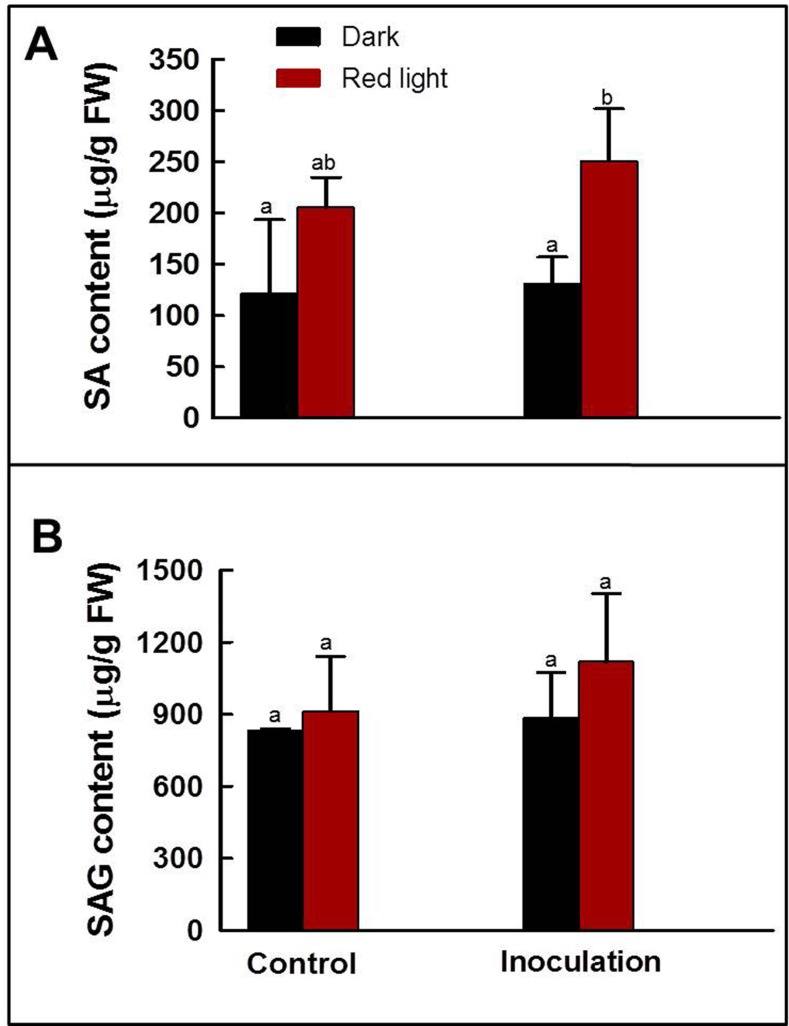
Content of free and conjugated salicylic acids. (A) Free salicylic acid (SA) and (B) glucose-conjugated salicylic acid (SA glucoside, SAG) in soybean sprouts grown in darkness or under red light irradiation. The SA and SAG contents of germinated soybean sprouts were measured without inoculation of *Pseudomonas putida* 229 (control), or after inoculation for 8 h followed by incubation in darkness for 24 h (inoculation). The experiments were repeated two times. The different letter indicates a significant difference (*P*<0.05).

To clarify the mechanism of resistance against the *P*. *putida* 229 in soybean sprouts germinated under red light, we measured the endogenous content of both free SA and SAG in soybean sprouts. Soybean sprouts germinated for 5 days in darkness or under red light were used as uninoculated controls or inoculated with *P*. *putida* 229 for 8 h and then subjected to incubation for 24 h in darkness (Fig. 3). The SA content of soybean sprouts germinated in darkness and under red light irradiation did not differ significantly (Fig. 3A). In contrast, the sprouts germinated under red light contained SA content more than 2.0 times greater than those germinated in darkness after *P*. *putida* 229 inoculation, representing a significant difference (Fig. 3A). The level of SAG in the soybean sprouts grown under red light was slightly higher than in soybeans grown in darkness; however, the level did not differ significantly before and after inoculation (Fig. 3B).

### JA contents in soybean sprouts irradiated with red light

**Fig 4:**
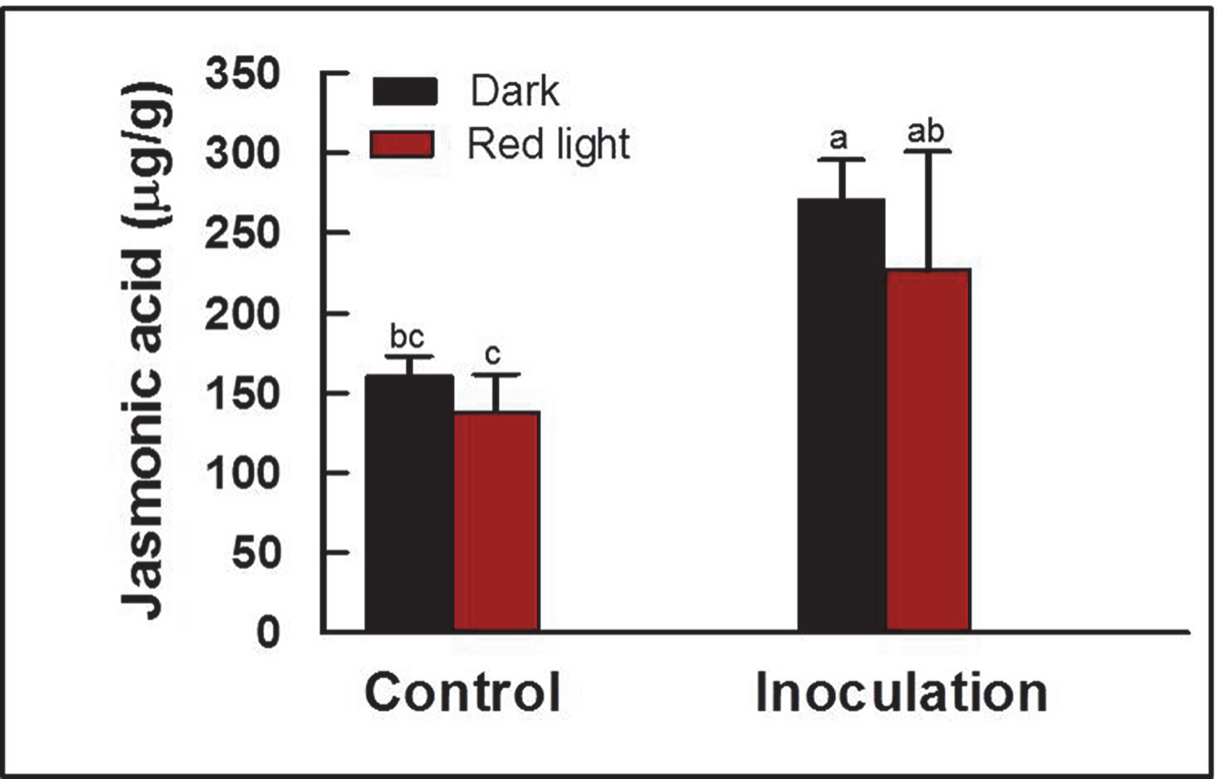
Content of jasmonic acids (JA). Content of JA in soybean sprouts germinated in darkness or under red light was analyzed. JA contents in the soybean hypototyls were measured without inoculation of *Pseudomonas putida* 229 (control), or with inoculation for 8 h followed by incubation in darkness for 24 h (inoculation). These experiments were repeated two times. Different letter indicates a significant difference (*P*<0.05).

The content of JA in soybean sprouts was determined to elucidate whether the disease resistance in the soybean sprouts germinated under red light was induced by JA-dependent pathway, though the expression patterns of *ACX* and *KAT* gene in the JA biosynthesis pathway implied that the resistance might not have been induced by high accumulation of JA. JA content was measured for the soybean sprouts germinated for 5 days in darkness or under red light as uninoculated samples, or for them inoculated with *P*. *putida* 229 for 8 h and then subjected to incubation for 24 h in darkness as inoculated samples (Fig. 4). JA content of uninoculated hypocotyls germinated in darkness or under red light was not significantly different. Both sprouts significantly increased the amount of JA after inoculation, however, each soybean sprout germinated in darkness or under red light did not differ the content of JA in the hypocotyls inoculated with *P*. *putida* 229 (Fig. 4).

## Discussion

Light is essential for the lives of plant species, providing energy for their survival and working as a signal to develop differentially and exert physiological changes. Great effort has been made to understand the roles of different light wavelengths on plant disease resistance [15,40,41]. Induced disease resistance against *Phytophthora capsici* has been observed in many plants, including pumpkin, pepper and tomato seedlings, in response to the application of red light treatment [15]. In broad bean, red light treatment suppressed the leaf spot disease caused by *Alternaria tenuissima* [40]. Irradiation of *Nicotiana benthamiana* with different wavelengths of light induced resistance against wildfire disease, especially by blue and red wavelengths [41].

Soybean yield is mainly decreased by the attack of several fungal, bacterial and viral diseases [42]. Several chemicals including 2,6-dichloroisonicotinic acid (INA), benzothiadiazole (BTH) and humic acids have been used to control these diseases [42,43]. Although these chemicals are effective at controlling such diseases, they can be expensive and exert adverse effects on the environment. Therefore, in this study, we used an environmentally friendly method that could be useful for identification of genes involved in resistance against disease infection. Furthermore, most studies of soybean have been conducted using seeds; therefore, investigations of the protection of soybean sprouts against disease infection are warranted. As shown in Fig. 1, red light irradiation increased resistance against *P*. *putida* 229 in soybean sprouts, which is in agreement with the results of earlier studies conducted using rice, *Arabidopsis* and broad bean [44–46]. The disease resistance induced by light was solely dependent on a specific range of wavelength, and intrusion of other wavelength might diminish the effect, as shown in the disease susceptibility in the soybean sprouts grown under the daylight fluorescence bulbs, which emitted mixed wavelength of red, green and blue area.

Plants develop resistance against pathogens by the induction of plant hormones such as SA, JA and ethylene [47]. The two major pathogen defense signaling pathways can be classified into (i) SA dependent pathway and (ii) SA-independent pathway that involves JA [29]. The signaling pathways triggered by SA and JA molecules are mutually antagonistic for the resistance against pathogens infection [29]. SA is well known for its important roles in pathogen resistance and SAR [48,49]. Mutants defective in SA synthesis showed increased susceptibility to powdery mildew [27]. In our study, the soybean sprouts germinated under red light contained 1.7 times higher SA contents than those germinated in darkness. When soybean sprouts were inoculated with *P*. *putida*, those germinated under red light accumulated significantly higher levels of SA (more than 2.0 fold) than those germinated in darkness (Fig. 3). These results suggest that SA-mediated defense responses are involved in the enhanced resistance to *P*. *putida* 229 in soybean sprouts germinated under red light. Similarly, cucumber plants treated with red light accumulated significantly higher SA levels only after *Sphaerotheca fuliginea* inoculation, however, no significant difference was observed when compared to red light irradiated and control plants without inoculation [50]. Previously, non-infected soybean leaves were shown to induce SAR activity via the SA-dependent pathway against *P*. *syringae* pv. glucinea through prior infection with *Phytophthora sojae* [51]. The non-significant expression levels of the *ACX*, *KAT* and *PR-4* gene but significant expression levels of the *ICS* and *PR-1* gene confirm that increased resistance is induced by the SA-dependent pathway. The involvement of solely the SA-dependent pathway for the induced resistance was confirmed by no difference between the content of JA in soybean sprouts germinated for 5 days in darkness or under red light (Fig. 4).

The SA-mediated defense was characterized by the endogenous synthesis of SA and activation of several *PR* genes encoding PR proteins. It has been proposed that SA production in plants occurs via the *ICS*-mediated pathway rather than the *PAL*-mediated pathway (Wildermuth et al. [38]. The increased levels of SA in soybean sprouts germinated under red light in the present study can be explained by up-regulation of the expression level of the *ICS* gene. The *ICS* gene in the soybean sprouts germinated under red light decreased the expression levels after 8 h of incubation, but these levels were recovered after 24 h of incubation in darkness. Moreover, the *ICS* gene expression level in soybean sprouts germinated under red light were significantly higher than those germinated in darkness after 24 h of incubation (Fig. 2A). The expression levels of the *ICS* gene in soybean sprouts germinated in darkness gradually decreased during the 24 h incubation period after the infection, which can be explained as a compatible interaction between the host plant and the pathogen that leads to a successful infection [52]. Indeed, the compatible relation as the successful infection was observed as rotting in 5 DAI for the soybean sprouts germinated in darkness (Fig. 1A). The recovered expression of the *ICS* gene in sprouts germinated under red light may imply the development of incompatibility in the sprouts in response to red light irradiation. In an incompatible interaction, plants gain resistance after pathogen infection by inducing different responses such as oxidative burst and SA accumulation [52,53], and our data clearly indicated that more SA accumulated in soybean sprouts germinated under red light irradiation (Fig. 3A). The expression levels of the *PR-1* and *PR-4* genes were also measured to determine the mechanism for the increased levels of disease resistance in soybean sprouts germinated under red light. *PR-1* gene expression levels were highly up-regulated by *P*. *putida* 229 inoculation in soybean sprouts germinated under red light, whereas *PR-4* gene expression levels were not changed (Fig. 2). *PR-1* is a molecular marker of the SA-dependent SAR pathway [48]; therefore, the high accumulation of the *PR-1* gene by *P*. *putida* infection suggests that the newly developed resistance in soybean sprouts germinated under red light is due to SAR by higher levels of SA.

Taken together, the expression levels of the *ICS* and *PR-1* gene and high levels of endogenous SA in soybean sprouts germinated under red light indicate that the resistance against the bacterial pathogen occurs via SAR. Further studies using molecular approaches and metabolome analysis could lead to identification of genes and associated biochemical pathways of key regulatory factors involved in the induction of resistance by red light irradiation.
